# Correlates and trajectories of relapses in relapsing–remitting multiple sclerosis

**DOI:** 10.1007/s10072-023-07155-3

**Published:** 2023-11-17

**Authors:** Carolyn A. Young, David J. Rog, Basil Sharrack, Radu Tanasescu, Seema Kalra, Timothy Harrower, Alan Tennant, Roger J. Mills, on behalf of the Trajectories of Outcome in Neurological Conditions-MS Study Group, Carolyn Young, David Rog, Basil Sharrack, Cris Constantinescu, Seema Kalra, Tahir Majeed, Helen Santander, Tim Harrower, Oliver Leach, Richard Nicholas, Helen Ford, John Woolmore, Chris Kipps, Clare Johnston, John Thorpe, David Paling, Yasser Falah, Cathy Ellis, Ashwin Pinto, C. Oliver Hanemann, Siddharthan Chandran, Andrea Malaspina, Jo Kitley, Jacqueline Palace, Tracy Fuller, Pat Mottram, Helen Terrett, Antonio Scalfari

**Affiliations:** 1 Walton Centre NHS Foundation Trust, Lower Lane, Fazakerley, Liverpool L9 7LJ, UK, University of Liverpool, Liverpool, UK; 2 Manchester Centre for Clinical Neurosciences, Northern Care Alliance NHS Foundation Trust, Salford, UK; 3 Academic Department of Neurology, University of Sheffield, Sheffield, UK; 4 University of Nottingham, Nottingham, UK; 5 University Hospital of North Midlands NHS Trust, Stoke-On-Trent, UK; 6 University of Exeter Medical School, Exeter, UK; 7 Leeds Institute of Rheumatic and Musculoskeletal Medicine, University of Leeds, Leeds, UK

**Keywords:** Multiple sclerosis, Relapse, Rasch, Patient-reported outcome measure, Trajectories of Outcome in Neurological Conditions-MS

## Abstract

**Background and aims:**

In people with relapsing–remitting multiple sclerosis (pwRRMS), data from studies on non-pharmacological factors which may influence relapse risk, other than age, are inconsistent. There is a reduced risk of relapses with increasing age, but little is known about other trajectories in real-world MS care.

**Methods:**

We studied longitudinal questionnaire data from 3885 pwRRMS, covering smoking, comorbidities, disease-modifying therapy (DMT), and patient-reported outcome measures, as well as relapses during the past year. We undertook Rasch analysis, group-based trajectory modelling, and multilevel negative binomial regression.

**Results:**

The regression cohort of 6285 data sets from pwRRMS over time showed that being a current smoker was associated with 43.9% greater relapse risk; having 3 or more comorbidities increased risk and increasing age reduced risk. Those diagnosed within the last 2 years showed two distinct trajectories, both reducing in relapse frequency but 25.8% started with a higher rate and took 4 years to reduce to the rate of the second group. In the cohort with at least three data points completed, there were three groups: 73.7% followed a low stable relapse rate, 21.6% started from a higher rate and decreased, and 4.7% had an increasing then decreasing pattern. These different trajectory groups showed significant differences in fatigue, neuropathic pain, disability, health status, quality of life, self-efficacy, and DMT use.

**Conclusions:**

These results provide additional evidence for supporting pwRRMS to stop smoking and underline the importance of timely DMT decisions and treatment initiation soon after diagnosis with RRMS.

**Supplementary information:**

The online version contains supplementary material available at 10.1007/s10072-023-07155-3.

## Introduction

A relapse in multiple sclerosis (MS) is a worsening of symptoms or the appearance of new neurologic symptoms, separated by at least 30 days from the onset of the last relapse and in the absence of fever or infection, which lasts at least 24 h and is followed by a period of partial or complete recovery [1]. In a study based on German Statutory Health Insurance data comprising 15,377 patients on disease-modifying therapy (DMT), with a mean age of 39.6 years (68% female), the overall rate of MS relapses per patient per year was 0.34 (95% CI 0.33–0.34) [2]. In an American patient-reported study using multiple online sources comprising 5311 people of whom 72.2% were diagnosed with relapsing–remitting MS (RRMS) and 74.8% were on DMT, with a mean age of 51.2 years (84.3% female), the annualized relapse distribution among all patients was 44.1% with < 1 relapse, 35.5% with 1–2 relapses, and 20.2% with > 2 relapses [3].

Follow-up analyses from the British Columbia database showed relapse rates to be age-related, peaking for those in their 3rd–4th decades [4]. New areas of gadolinium enhancement are considered surrogate markers for relapses [5] and similar age-related patterns have been found with gadolinium-enhancing lesions, with an almost linear decrease in the percentage of subjects with enhancing lesions with increasing age [6]. A simulation study found a substantial age-specific decrease in annualized relapse rates, independent of disability worsening [7]. Under a range of clinically plausible assumptions, 88–97% of the decrease was attributed to age and only 3–13% to disability. Another study based upon 9705 people with MS found that the risk of relapses decreased continuously to about 35 years of age. Relapse risk remained stable for about a decade and then again continuously decreased [8].

There is debate over factors other than age that may influence relapse rates. A retrospective study of 251 MS subjects calculated their Framingham risk score reflecting age, sex, diabetes, smoking, systolic blood pressure, and body mass index. Over 5 years, a 1-point increase in the Framingham risk score was associated with a 31% higher risk of relapse [9]. In a study of 885 participants, those with migraine, hyperlipidemia, or a high comorbidity burden (3 or more conditions) were found to have an increased relapse rate over 2 years [10]. Conversely, another study of 1008 patients belonging to a trial population found that migraine, dyslipidemia, and a number of comorbid conditions were not associated with relapse activity [11]. A clinic-based prospective study of 230 people with RRMS found that comorbidity was not associated with relapse rate [12]. Finally, a recent review found the relationship between air pollution and relapse rate to be uncertain [13].

This study will explore the correlates of MS relapses with a wide range of socio-demographic and clinical variables, such as age, gender, education, air pollution and other deprivation indices, smoking status, and comorbidities, as well as examine relapse rate trajectories.

## Methods

### Samples

#### Main sample

Participants were recruited from 2013 to 2019 into the Trajectories of Outcome in Neurological Conditions-MS (TONiC-MS) study where eligibility criteria included adults with MS (by McDonald criteria [14]) of any disease subtype and level of disability. Data on disease subtypes at the time of study entry were provided by clinicians involved in the patients’ care. Participants with RRMS were split into two groups: those classified as rapidly evolving (RE) RRMS, if they had experienced two or more disabling relapses in the past year and brain magnetic resonance imaging (MRI), if repeated, showed one or more gadolinium-enhancing lesions or a significant increase in T2 lesion load as compared with previous recent MRI, and the remainder of RRMS not fulfilling these activity criteria, hence termed RRMS. The following analysis is based upon adults who entered the study with either RRMS or RE RRMS, as confirmed by a clinician.

Duration since diagnosis and Expanded Disability Status Scale (EDSS) band were recorded from the medical records as was the use of disease-modifying therapy (DMT) [15]. DMT was divided into “low efficacy” and “high efficacy” compounds [16]. Informed consent was obtained from all participants prior to enrolment. All data in this analysis was collected by the end of 2019, to avoid any possible effect of the SARS-CoV-2 pandemic on relapses and their reporting or management. Ethical approval was granted from research committees (reference 11/NW/0743).

From within this main sample, three further samples were drawn to facilitate the understanding of the trajectories of relapses:

- *Inception cohort—*comprising those who had been diagnosed with RRMS or RE RRMS less than 2 years before study entry.
- *Trilogy cohort—*comprising those who had completed at minimum the baseline and the first two follow-up questionnaires, over approximately 18 months.
- *Regression cohort—*comprising a subset of the main sample with data on comorbidities. This was not available for the entire main sample because comorbidities were not asked in the pilot study.

### Patient-reported outcome measures

Participants completed questionnaire packs containing a number of patient-reported outcome measures (PROMs) listed below. Initial completion is referred to as the “baseline.” Follow-up questionnaire packs were then sent at approximately 9-month intervals.

- *Number of relapses—*a simple count of the number of self-reported relapses during the previous year, henceforth described as annual relapse rate (ARR).
- *Disability: WHODAS2.0* [17]*—*32-item version with a range of 0–128.
- *Health Status: EQ-5D-5L—*EQ-5D-5L utility value derived from 5 items scored 1–5; the range is from − 0.285 to 1, where higher scores indicate better health states [18] [19]. Age-sex normative values have recently been published [20].
- *Fatigue: Neurological Fatigue Index-MS (NFI-MS)—*10-item summary scale scored 0–30, where higher scores represent greater MS fatigue [21].
- *Neuropathic Pain: Neuropathic Pain Scale—*consisting of 10 items scored 0–10 [22].
- *Self-Efficacy: Unidimensional Self-Efficacy Scale for MS (USE-MS)—*12 items scored 0–3, reflecting confidence to complete tasks and produce desired outcomes [23].
- *Quality of Life (QOL): World Health Organization Quality of Life Scale-BREF (WHOQOL-BREF)—*24 items covering four domains: physical, psychological, and social relationships and environment. Two stand-alone questions on QOL and satisfaction with health are not included. A total score from the 24 items, obtained from a bi-factor solution, was used in this analysis [24].

All ordinal scores from the PROMs were transformed to interval scaling via the Rasch model through previously derived transformation tables [25]. Full details of the methodology are given in the Online Resource, Sect. 1.1.

In addition to this, an index of air pollution was included from the UK national deprivation indices [26]. The indicator is an estimate of the concentration of the four pollutants nitrogen dioxide, benzene, sulfur dioxide, and particulates. For each pollutant, the atmospheric concentration in each area was compared to a national standard value. Comorbidities were self-reported in an additional comorbidity questionnaire which has been validated for self-report in people with MS [27]. This was included after the pilot study.

### Statistical analysis

After descriptive statistics, the potential risk factors affecting the relapse rate highlighted in the introduction were explored through a multilevel negative binomial regression. The relapse counts in the year preceding baseline and each follow-up period were included.

Using the different cohorts described above, the analysis proceeded to a group-based trajectory model to ascertain if there are groups displaying different relapse trajectories over time [28]. Duration was included as a covariate in the trilogy cohort. Full details of the methodology are given in the Online Resource, Sect. 1.2.

## Results

### Samples

The full sample consisted of 6388 responses over a number of time points. This comprised 3843 at baseline, 1543 at first follow-up, 712 at second follow-up, and 290 subsequently. The time between the baseline and first follow-up was 23.0 months, and between the first and second follow-up, 10.9 months. The data were formatted in a long (stacked) format, that is, the same person entered more than once when they completed more than the baseline. At the study entry, the mean number of relapses over the previous year was 0.48 (95% CI: 0.45–0.51), falling to 0.29 23 months later. The incidence risk ratio (IRR) of relapse at the first follow-up was 66.5% (CI: 36.2–53.4) of that at baseline, adjusted for duration less than 2 years, and age.

The main cohort consisted of 3885 people with RRMS, where data were formatted in a wide (racked) format such that there was one line per person. Their characteristics at baseline are shown in Table 1. The health status utility was just below the normative values for that age group. Of note, those with an EDSS level of ≤ 4 had utility values slightly higher than the norms. Just under one in eight (11.7%) reported having two or more relapses in the previous year. Just over one in nine (11.4%) had an EDSS < 4.5 with a minimum of 15-year duration since diagnosis, defined as benign MS in the current study; those with benign MS were less likely (53.7%) to be on DMT than the remainder (60.4%) (*χ*^2^ 7.19 (df1); *p* = 0.007). Those with RE RRMS, comprising just over one in sixteen (6.36%) of all RRMS, were much more likely (86.6%) to be on DMT, compared to those whose RRMS did not meet that activity criterion at study entry (57.1%) (*χ*^2^ 79.9 (df 1); *p* ≤ 0.001). There was no significant difference in mean number of relapses over the previous year (ARR) for gender (t 0.544 (df 1); *p* = 0.586).

**Table 1:** Baseline demographic characteristics of main, inception, and trilogy cohorts

Characteristic	Main	Inception	Trilogy
*n*	3885	666	695
Mean age/years (SD)	46.0 (10.9)	40.6 (10.9)	47.5 (10.9)
Mean duration since diagnosis/years (SD)	9.0 (8.4)	0.62 (0.49)	9.2 years (8.7)
% female	77.4	76.4	80.9
Current smoker	11.9	13.4	8.2
EDSS bands^a^ as %			
0–4	70.5	83.5	67.1
4.5–6.5	27.3	15.3	31.2
≥ 7	2.2	1.2	1.7
% on DMT^b^	59.6	42.3	59.0
- Moderate	39.1	29.1	39.8
- High	20.5	13.2	19.2
% working	60.1	79.6	61.7
% medically retired	18.0	5.9	18.4
Health utility value	0.759	0.773	0.788
Relapses reported as %			
None	69.4	51.1	72.2
1	18.8	24.6	16.5
≥ 2	11.8	24.3	11.3
Mean number of relapses at baseline over the previous year—ARR^c^	0.48 (95% CI^d^: 0.45–0.51)	0.88 (95% CI: 0.79–0.98)	0.43 (95% CI: 0.37–0.49)

^*a*^*EDSS* Expanded Disability Status Scale; ^b^*DMT* disease-modifying therapy; ^c^*ARR* annual relapse rate; ^d^*CI* confidence interval

Overall, 59.6% of the main cohort was on DMT, with males having a higher proportion (64.3%) than females (58.3%) (*χ*^2^ 10.3 (df 1); *p* = 0.001). Those who were relapse-free were marginally more likely to be on DMT (60.9%) than those experiencing one or more relapses (56.9%) (*χ*^2^ 5.62 (df 1); *p* = 0.018). Those in paid work were more likely to be receiving DMT (61.2%) than those not (57.1%) (*χ*^2^ 6.37 (df 1); *p* = 0.012). Of those working aged between 25 and 50, 56.8% of females and 67.7% of males received DMT (*χ*^2^ 18.4 (df 1); *p* ≤ 0.001). The odds ratio for receiving DMT when a non-working female was aged between 25 and 50 years was 0.61 (95% CI: 0.48–0.76) compared to working males of the same age, adjusted for duration (*p* ≤ 0.001). However, there was no significant difference in the ARR between working males and non-working females (*t* = 1.06 (df 1707); *p* = 0.286). Also, the ARR for those on DMT was 0.46, while for those not on DMT was 0.51, which was not significantly different (*t* = 1.66 (df 3841); *p* = 0.098).

The inception cohort of 666 people with recently diagnosed RRMS had a greater proportion with EDSS 0–4 (83.5%) compared to the remainder in the main cohort (67.8%) (*χ*^2^ 65.1 (df 1); *p* < 0.001) but had an ARR approaching twice that of the overall cohort (see Table 1). Just over two-fifths (42.3%) were on DMT. Over three-quarters (79.6%) were in work.

The trilogy cohort consisted of 695 people with RRMS (pwRRMS) who had completed their first three questionnaires. Females were slightly more likely to provide trilogy data (an increase of 3.5% above their original 77.4%) compared to males (a decrease of 3.5% out of their original 22.6%) (*χ*^2^ 6.10 (df 1); *p* = 0.013). Almost a third (32.9%) were in EDSS band 4.5 and above, and 59.0% were on DMT (see Table 1).

### Regression

A multilevel negative binomial regression on the regression cohort of 6285 data sets from pwRRMS over time, with a number of relapses as the dependent variable, revealed that the factor having the most influence to increase relapse risk was being a current smoker compared to having never smoked, which raised the risk by 43.6% (Table 2). Being a past smoker compared to never having smoked did not significantly increase risk. Having three or more comorbidities also weakly increased the risk of relapses, compared to less than three comorbidities. In contrast, DMT reduced the risk by 18%. Despite the overall impact of DMT in risk reduction, there was no significant difference in the number of relapses between either moderate- or high-efficacy DMT (*t* = 1.86 (df 3801); *p* = 0.063). Age also reduced the risk. Hyperlipidemia, diabetes, and migraine had no significant effect along with all the deprivation indices (not shown), including air pollution. No significant effects on the risk of relapses were found for the duration, gender, or education level.

**Table 2:** Multi-level negative binomial regression with dependent variable the number of relapses. *n* = 6285

Characteristics	IRR^a^	St. err	*t*-value	*p*-value	[95% conf. interval]		Sig
*Clinical*							
3 + comorbidities	1.162	.087	2.01	.044	1.004	1.346	**
Duration	.995	.004	− 1.18	.247	.987	1.003	
DMT	.821	.051	− 3.17	.002	.726	.928	***
*Demographics*							
Age	.965	.003	− 11.17	0	.959	.971	***
Female gender	.98	.073	− 0.27	.786	.846	1.135	
*Personal factors*							
Smoking status:							
- Past	1.018	.069	0.26	.792	.891	1.164	
- Current	1.436	.135	3.86	0	1.195	1.725	***
Educational status	1.082	.076	1.12	.262	.943	1.243	
*Environmental factors*							
Air quality	.987	.06	− 0.22	.825	.876	1.111	
Constant	1.639	.275	2.95	.003	1.18	2.277	***

^a^*IRR* incidence risk ratio > 1 = higher risk of relapses, < 1 = lower risk; ***p < .01, **p < .05, *p < .1; model fit: chi-square 188.1 *p* =  < 0.001

### Trajectory analysis

#### Inception group (N = 666)

**Fig. 1:**
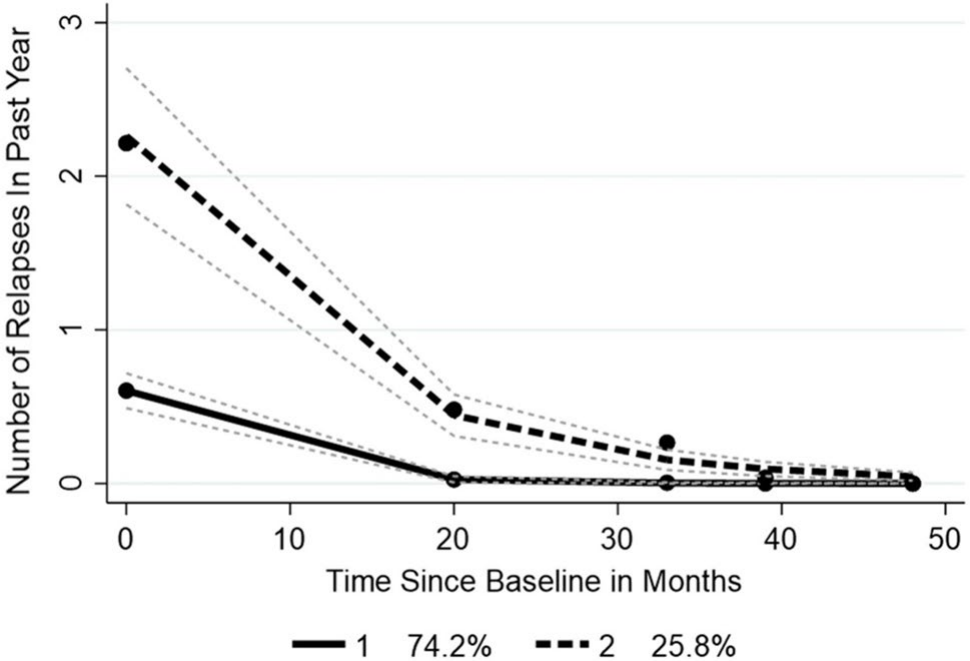
Relapse trajectories for the inception cohort. *n* = 666. Fine dashed lines indicate 95% confidence limits

Two groups were identified with significantly different ARRs; the larger group 1 (*n* = 494) with an ARR of 0.409 (SD 0.027) and group 2 (*n* = 172), with about one-quarter of the cohort, showing an ARR of 2.47 (SD 0.114) (*t*-test 25.98 (df 664); *p* ≤ 0.001). While both groups showed a decline in relapses over 4 years, the slopes of each group were significantly different, so it took 4 years for the number of relapses in group 2 to fall to the level of group 1 (Fig. 1). The two groups did not differ by duration (*t* = 0.122 (df 664); *p* = 0.903) nor was the proportion with an EDSS ≥ 4.5 different between groups with group 1 at 15.9% and group 2 at 18.5% (*χ*^2^ 0.56 (df 1); *p* = 0.451). However, the use of DMT differed significantly between groups, with 39.7% for group 1 and 50.0% for group 2 (*χ*^2^ 5.6 (df 1); *p* = 0.018).

Health status and QOL differed significantly across groups at baseline with group 2 showing worse outcomes on all aspects except gender, EDSS, and duration, as well as having a slightly higher but not significant proportion of those with the subtype RE RRMS (Table 3).

**Table 3:** Baseline health status and quality of life characteristics of inception and trilogy trajectory groups

Characteristic	Inception cohort		Trilogy cohort			Range^a^
*Groups*	1	2	1	2	3	
*N*	494	172	512	150	33	
%	74.2	25.8	73.7	21.6	4.7	–
*Demographics*						
Age/years	41.6	37.7	48.9	42.3	49.3	17–87
% female	75.9	77.9*	81.1	80.7	84.8*	–
Duration/years	0.62	0.61*	10.5	4.6	9.5	0–67
Current smoker	11.9	17.4*	7.8	9.3	12.1*	0–100
*Patient-reported outcome measures (PROMs)*						
Fatigue^b^	15.4	17.7	16.5	18.8	20.2	0–36
Neuropathic pain^c^	35.9	39.7	38.2	40.6	41.7	0–100
Disability^d^	27.7	36.0	31.1	35.6	42.1	0–128
Health status^e^ ^	0.803	0.743	0.787	0.740	0.685	− 0.28–1
Quality of life^f^ ^	55.6	51.2	54.6	50.8	46.0	0–96
Self-efficacy^g^ ^	20.9	18.7	20.5	18.7	18.3	0–36
*Clinical factors*						
% EDSS ≥ 4.5	15.9	18.5*	34.0	26.0	48.5	0–100
% on DMT^h^	39.7	50.0	60.9	57.3	54.6*	0–100
% rapidly evolving RRMS^i^	6.1	7.6*	5.5	8.0	6.1*	0–100
Annual relapse rate	0.41	2.47	0.08	1.4	1.5	0–∞

All group comparisons are significant (*t*-test; ANOVA; *χ*^2^ *p* =  < 0.05) unless indicated by *

^High score good. ^a^Range: for PROMs the full operational range of the scale. ^b^Neurological Fatigue Index-MS. ^c^Neuropathic Pain Scale. ^d^WHODAS 2.0–32-item version. ^e^EQ-5D-5L utility value. ^f^World Health Organization Quality of Life Scale-BREF. ^g^Unidimensional Self-Efficacy Scale for MS. ^h^Disease-modifying therapy. ^i^Relapsing-remitting multiple sclerosis

#### Trilogy group (N = 695)

**Fig. 2:**
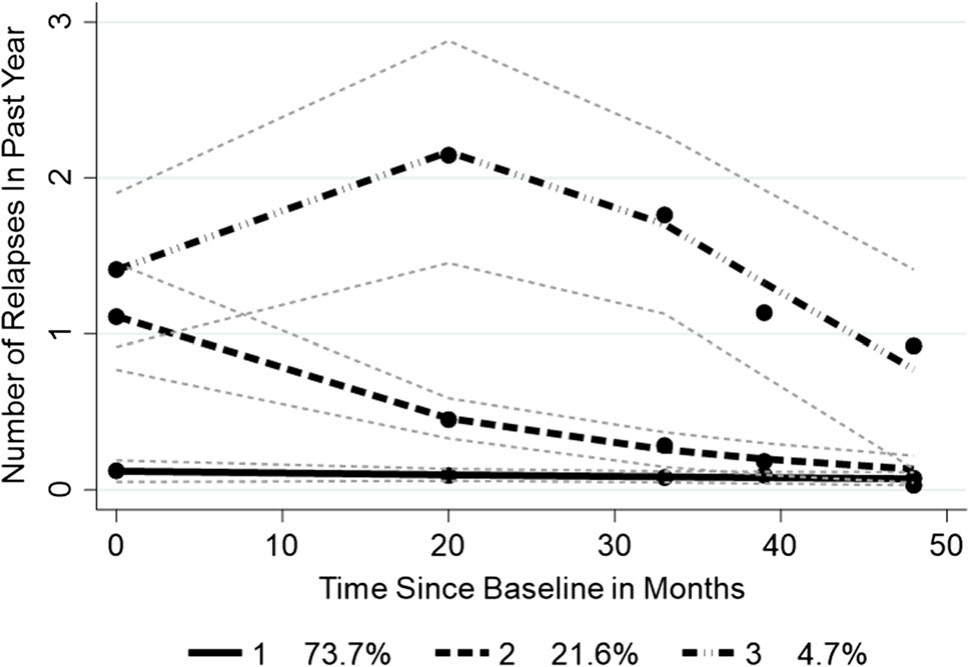
Relapse trajectories for trilogy cohort. *n* = 695. Fine dashed lines indicate 95% confidence limits

Three groups were identified (Fig. 2). The intercept of group 1 is significantly different from groups 2 and 3 and accounts for almost three-quarters of cases. It has a flat non-significant trajectory of relapses over the 4 years. In contrast, group 2 shows a significant declining relapse trajectory, while group 3 has a non-linear rising and declining trajectory. The ARR was 0.08 for group 1, 1.4 for group 2, and 1.5 for group 3. Note that group 2 has a much shorter duration than groups 1 and 3. There was no significant difference in DMT use across groups (*χ*^2^ 1.04 (df 2); *p* = 0.596). The effect size of the baseline difference in duration between groups 1 and 2 is 0.29, considered small (*F* 4.75 (df 2, 653); *p* = 0.009). There is a deteriorating gradient of health status and QOL across groups 1 to 3 (Table 3). Although the latter group is small, it is interesting as it consists of predominately older females, with a long disease duration, maintaining an ARR of 1.5 with over half (54.6%) on DMT. Almost all (91.0%) of those considered benign in the current study were in group 1 (*χ*^2^ 15.17 (df 2); *p* = 0.001).

## Discussion

In the main sample of 3885 pwRRMS, ARR was 0.48 overall, with an increased risk of relapses in current smokers and those with multiple comorbidities, and reduced risk with DMT use and increasing age. Previous findings relating to the effects upon relapses of age [4] and 3 or more comorbidities [10] have been replicated in this study but not earlier findings on increased relapse rate with migraine or hyperlipidemia [10]. We did not find air pollution to be a significant predictor of the number of relapses.

Recent reviews have described the effect of current smoking on the risk of relapse as “contradictory” [29] or “unknown” [30] but our results clearly showed a significantly increased risk of 43.6% compared to having never smoked, but not significantly increased risk for past smokers, strengthening the need to support people with MS to stop smoking. Future investigations should also consider the impact, if any, of vaping.

The overall ARR conceals very distinct trajectories, the identification of which reveals important clinical messages. The analysis of the different cohorts is important for the specific information provided; the inception cohort examines relapse rate and risk factors in people recently diagnosed with RRMS or RE RRMS. It transpired that the inception cohort, with an average duration since diagnosis of just over 7 months, already showed two distinct groups. Although people in the group with higher baseline ARR (2.47) were significantly more likely to receive DMT, only 50.0% were on DMT at the time of data collection, suggesting scope for improvement in the timely provision of DMT for people with MS. While both groups showed falling relapse rates, the group showing higher baseline ARR, consisting of almost a quarter of the inception cohort, took 4 years to reduce to the level experienced by those in the other group and showed worse health status and QOL from baseline.

In the main cohort of 3885 people with RRMS, 59.6% overall were on DMT; those with RE RRMS were more likely to be on DMT (86.6%). The use of DMT in this large sample showed unexpected differences according to demographic characteristics such as a greater proportion having DMT if males in paid employment, compared to females without employment, where the odds ratio for females was just 0.61. Consequently, the probability that such females will not receive DMT, given an equal ARR to working males and aged between 25 and 50, is 39%. Logistic regression (not shown) shows male gender to be a significant predictor of DMT prescription, whereas employment is not, adjusted for disability level (EDSS) and age. This gives prima facia evidence that there is gender bias in prescription in our large sample. This could reflect socio-economic influences or awareness of faster disability accumulation in male patients with RRMS [31].

The trilogy cohort was studied to strengthen the analysis of non-linear trajectories. Results showed three trajectories: one with a non-linear increasing and then decreasing trajectory (4.7%), one with a falling relapse rate (21.6%), and one large group with virtually no relapses over 4 years (73.7%). There was no significant difference in DMT use across groups. However, these three groups showed significant differences in fatigue, neuropathic pain, disability, health status, quality of life, and self-efficacy; having a low stable relapse rate was the most favorable for all the above factors followed by having a falling relapse rate. While the group showing the non-linear rising and then falling relapse rate was small (4.7%), it is a clinically important group, which may have been misinterpreted as a slow declining group if only two data points at baseline and 4 years had been collected. Consisting of predominately females, this group maintained a high level of relapses, despite a comparable use of DMT to the low stable relapse group, and this was associated with the worst health status and QOL.

An important response to the findings in the current study is to focus on reducing relapses in the early stage of the disease. The inception cohort analysis indicated that while EDSS levels were similar between groups, this was not the case for the ARR. No socio-demographic or PROM data could differentiate between groups (not shown), and so it would seem that any patient presenting to the neurologist for diagnosis, who has experienced more than one relapse in the previous 12 months, would be a candidate for the higher activity group, with an increased risk of poorer health status and QOL. In the future, personalized medicine may allow the prediction of those at greater risk of relapse [32]. In current clinical practice, strong efforts to support people with RRMS to stop smoking, and facilitating timely access to DMT, alongside support to adhere to treatment, are clearly important.

There are several strengths to this study, such as its large size and national recruitment across the UK, longitudinal follow-up with multiple data points to detect non-linear trajectories, comprehensive interval-level PROMs, and trajectory analyses. While the main sample may be considered representative of those attending the various clinics involved in the study, both the inception cohort and the trilogy cohort results may have been biased by attrition at follow-up. However, the analysis did adjust for differential trajectory dropout. Despite the difference in the ARR between baseline and first follow-up, the effect size of that difference was 0.22, considered small. Furthermore, the relapse rate would be expected to fall over time, particularly in the inception cohort, which would contribute to the overall fall of the rate over time.

Not knowing the background to DMT use is also a limitation, such as if people not on DMT were planning pregnancy, were waiting for an initiation date, had recently discontinued DMT, or changed DMT during the course of the study, or were not engaging with MS services.

The observation that some people with MS experienced a low and others a higher ARR shortly after diagnosis suggests that further studies should investigate the pre-diagnosis period. Analyses of ambulatory claims data suggested that many of the symptoms recorded more frequently in patients with MS in the years before first diagnosis could represent demyelinating events that have not been recognized as such [33]. Future studies may include information from their earlier medical contacts, through their first contact with a neurologist, until diagnosis and beyond, which would seem crucial to a better understanding of the early pattern of relapses. The possible effect of high-efficacy DMT on trajectories warrants further detailed investigation. In addition, future studies should link genetic variation to the lived experience of MS, not just relapses but the whole continuum of experience as defined in the Wilson and Cleary model [34].

While the study found an overall ARR of 0.48, this masked considerable difference, not only by cohort, where, for example, the ARR of the inception cohort was almost twice as high, but also by those following different trajectories over time in the same cohort. The data raise questions about the equity of access to DMTs, where non-working females seemed to be at a disadvantage compared to their working male counterparts of the same age. Previous evidence of the effect of age was supported by this study, but earlier work on factors such as migraine, hyperlipidemia, and air pollution was not reproduced. Conflicting findings from earlier smaller studies on comorbidities and smoking were resolved in this study of over 3000 pwRRMS. These results provide additional evidence for supporting pwMS to stop smoking, as being a current smoker is associated with over 43% higher relapse risk. The importance of timely DMT decisions and treatment initiation soon after diagnosis with RRMS, as well as supporting adherence, are highlighted.

### Supplementary information

Below is the link to the electronic Online resource.

Online resource (PDF 181 KB)

## Data Availability

Data Access Data supporting this study are not openly available due to reasons of sensitivity and are available from the corresponding author upon reasonable request. Data are located in controlled access data storage at Walton Centre NHS Trust. Please contact wcft.tonic@nhs.net.
